# Supplementary material for: A Yes-Associated Protein (YAP) and Insulin-Like Growth Factor 1 Receptor (IGF-1R) Signaling Loop Is Involved in Sorafenib Resistance in Hepatocellular Carcinoma
Source: Cancers (Basel). 2021 Jul 29;13(15):3812. doi: 10.3390/cancers13153812 (PMC8345119; doi:10.3390/cancers13153812)
Supplement: Supplementary file 1 [file cancers-13-03812-s001.zip › cancers-1262667-supplementary.pdf]

cancers

Supplementary Materials

A Yes-Associated Protein (YAP) and Insulin-Like Growth Factor 1 Receptor (IGF-1R) Signaling Loop Is Involved in Sorafenib Resistance in Hepatocellular Carcinoma

Mai-Huong T. Ngo, Sue-Wei Peng, Yung-Che Kuo, Chun-Yen Lin [4], Ming-Heng Wu, Chia-Hsien Chuang, Cheng-Xiang Kao, Han-Yin Jeng, Gee-Way Lin, Thai-Yen Ling, Te-Sheng Chang, and Yen-Hua Huang

Citation: Ngo, M.-H.T.; Peng, S.-W.; Kuo, Y.-C.; Lin, C.-Y.; Wu, M.-H.; Chuang, C.-H.; Kao, C.-X.; Jeng, H.-Y.; Lin, G.-W.; Ling, T.-Y.; et al. A Yes-Associated Protein (YAP) and Insulin-Like Growth Factor 1 Receptor (IGF-1R) Signaling Loop Is Involved in Sorafenib Resistance in Hepatocellular Carcinoma. *Cancers* **2021**, *13*, 3812. https://doi.org/10.3390/cancers13153812

Academic Editor(s): Hiroshi Nishina

Received: 31 May 2021
Accepted: 22 July 2021
Published: 29 July 2021

Publisher's Note: MDPI stays neutral with regard to jurisdictional claims in published maps and institutional affiliations.

Copyright: © 2021 by the authors. Licensee MDPI, Basel, Switzerland. This article is an open access article distributed under the terms and conditions of the Creative Commons Attribution (CC BY) license (http://creativecommons.org/licenses/by/4.0/).

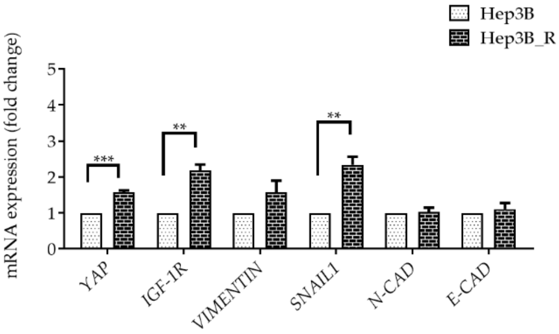

Figure S1. The mRNA expression levels of YAP, IGF-1R, and EMT-markers in naïve/resistant Hep3B cells. Data are the mean ± SEM of at least three independent experiments. $^{**}p < 0.01$ and $^{***}p < 0.001$ by Student's t-test.

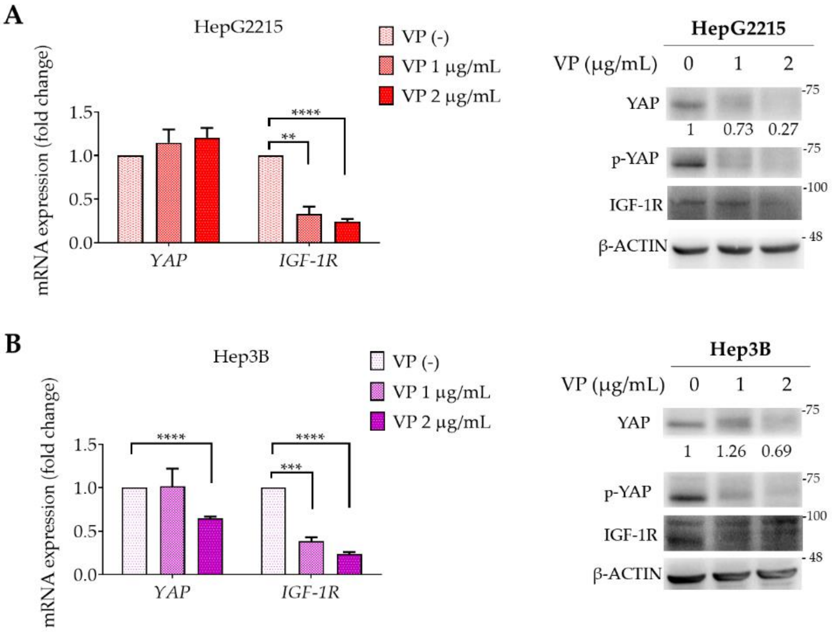

Figure S2. Effect of VP (a specific YAP inhibitor) on the mRNA and protein expression levels of YAP and IGF-1R in (**A**) HepG2215 cells and (**B**) Hep3B cells. Data are the mean ± SEM of at least three independent experiments. $^{**}p < 0.01$, $^{***}p < 0.001$, and $^{****}p < 0.0001$ by Student's t-test. The relative quantification was normalized to the corresponding β-ACTIN.

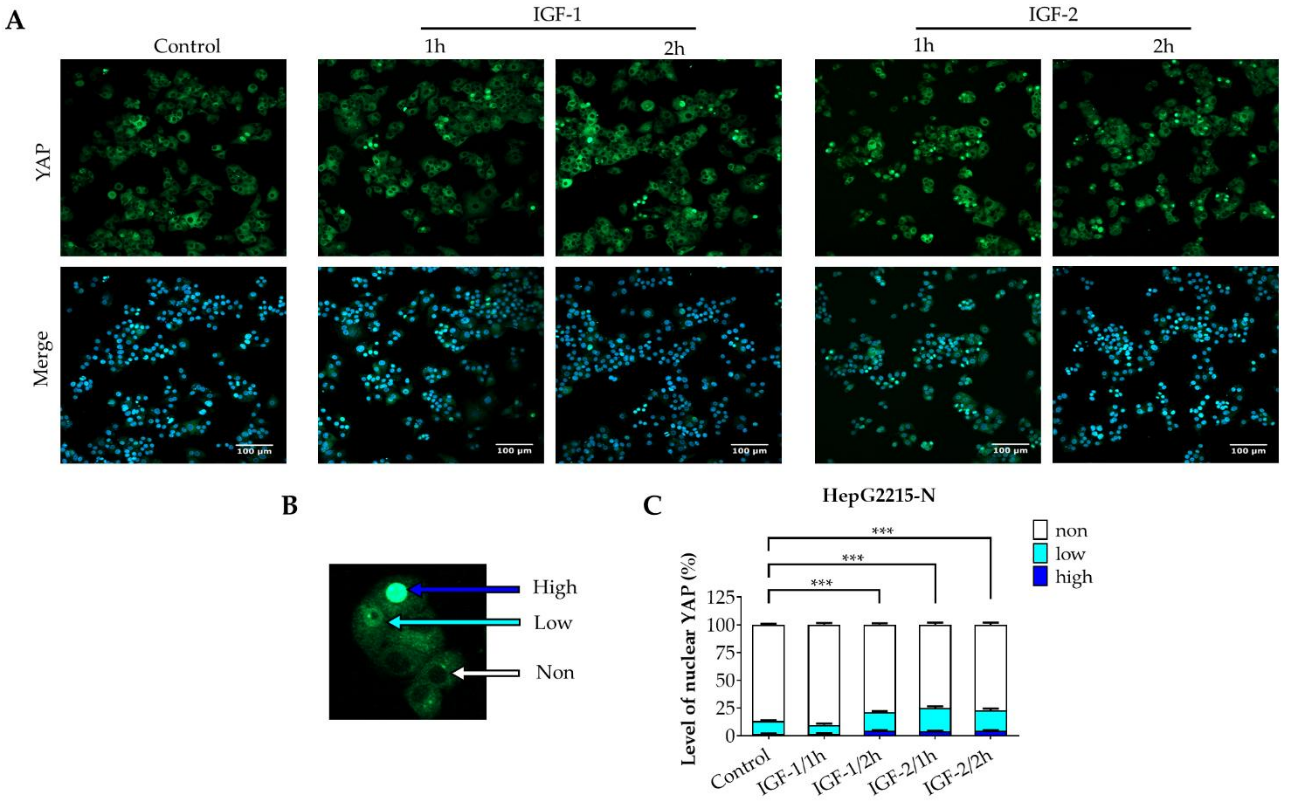

Figure S3. IGF-1R activation induces YAP nuclear translocation in HepG2215 cells. (**A**) The effect of IGF-1/2 (50 ng/mL, 1 h or 2 h treatment) on YAP nuclear translocalization in HepG2215 cells; (**B**) the expression level of nuclear YAP in HepG2215 cells was divided into three groups based on the fluorescence intensity; (**C**) the quantitative analysis of (**B**). Data are the mean ± SEM of at least eight random image fields of (**B**) for each group. ***$p < 0.001$ by Student's *t*-test.

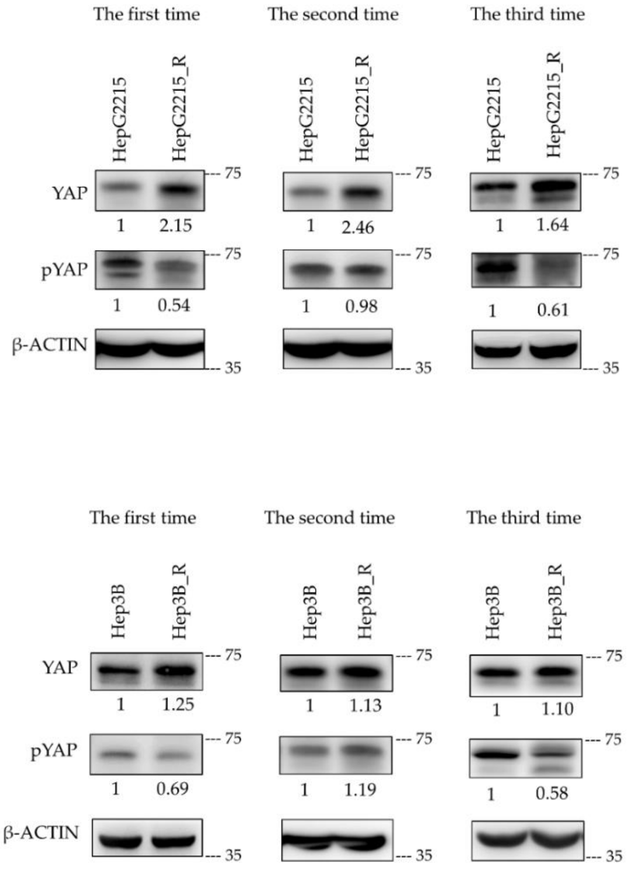

Figure S4. The triple repeats of Western blot and densitometry readings/intensity ratio of each band of main Figure 1D. The protein levels of YAP and p-YAP in HepG2215/HepG2215_R and Hep3B/Hep3B_R cells were shown. The relative quantification was normalized to the corresponding β-ACTIN.

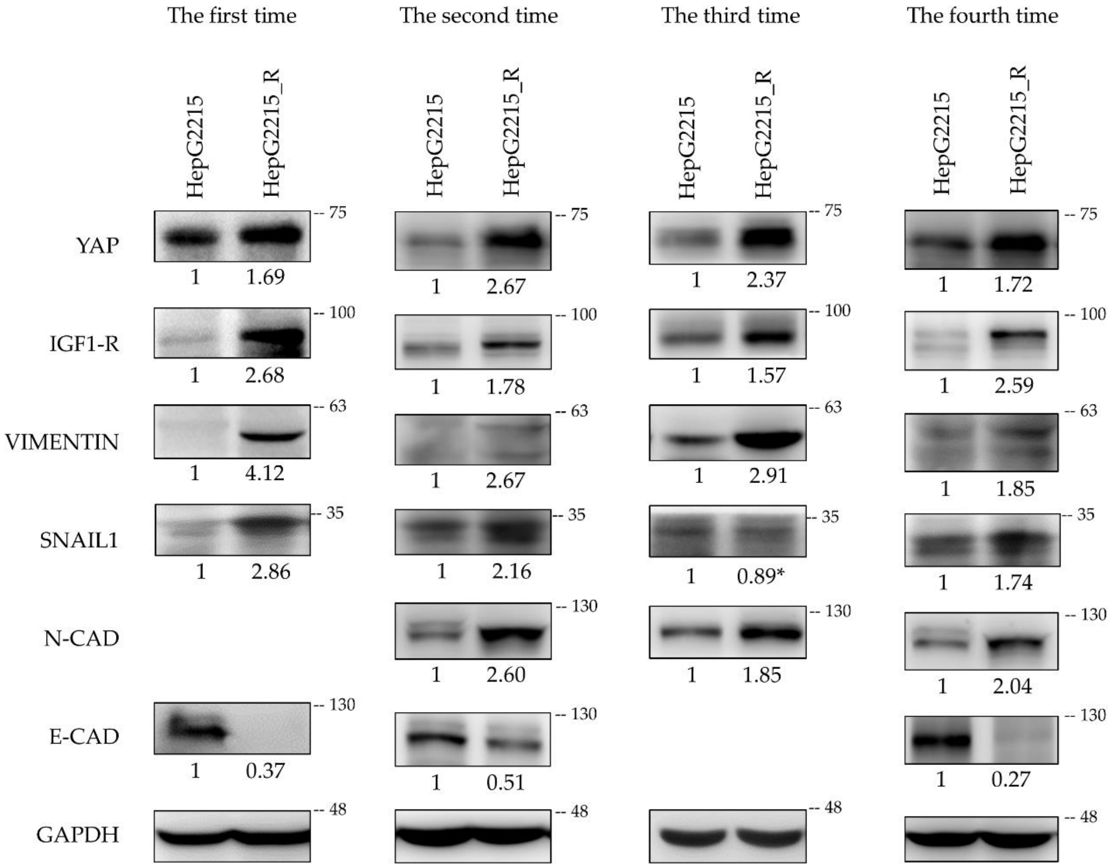

Figure S5. The four repeats of Western blot and densitometry readings/intensity ratio of each band of Figure 2B. The expression levels of protein of YAP, IGF-1R and EMT markers in naïve/resistant HepG2215 and HepG2215_R cells. The relative quantification was normalized to the corresponding GAPDH.

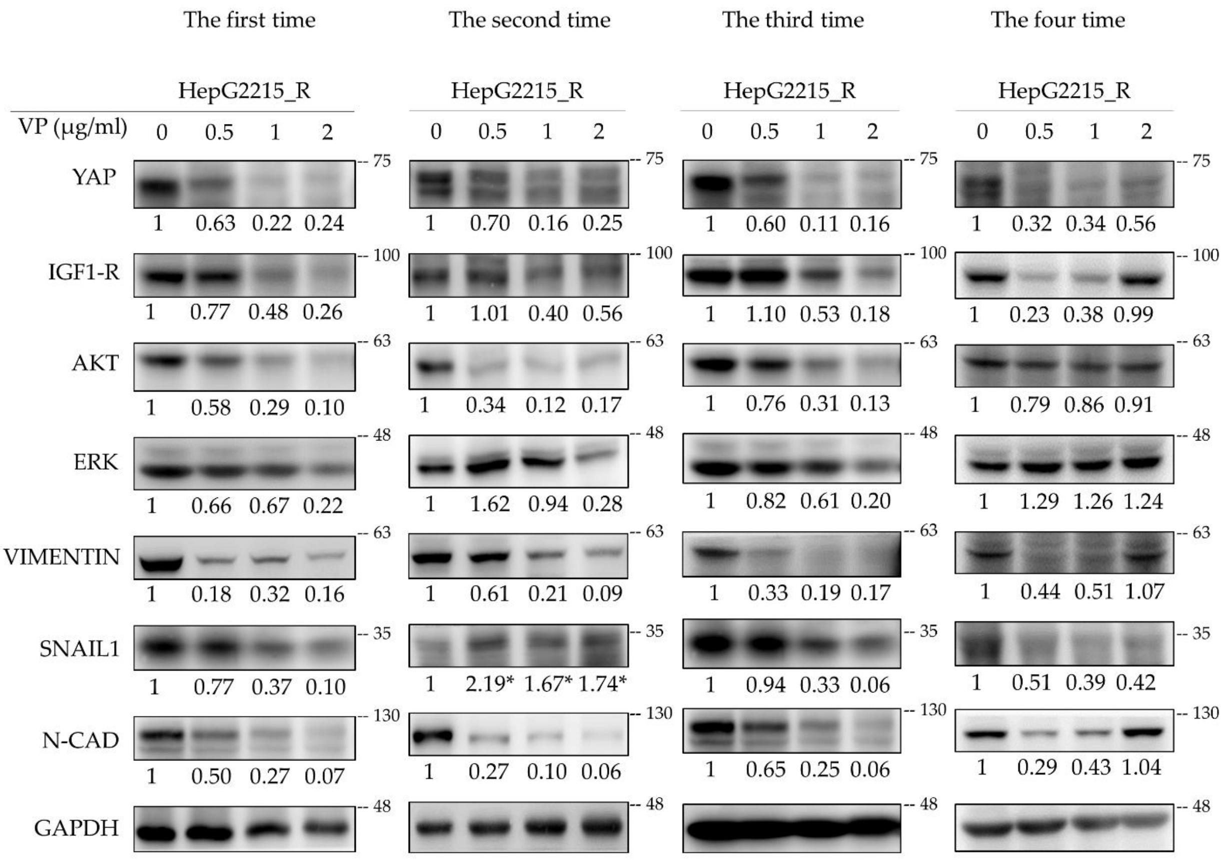

Figure S6. The four repeats of Western blot and densitometry readings/intensity ratio of each band of Figure 3B. The effect of VP (a specific YAP inhibitor) on the expression levels of protein of YAP, IGF-1R signaling-related proteins, and the EMT-markers in HepG2215-R cells. (*) The data was not included in the Student's t-test in Main Figure 3C(a). The relative quantification was normalized to the corresponding GAPDH.

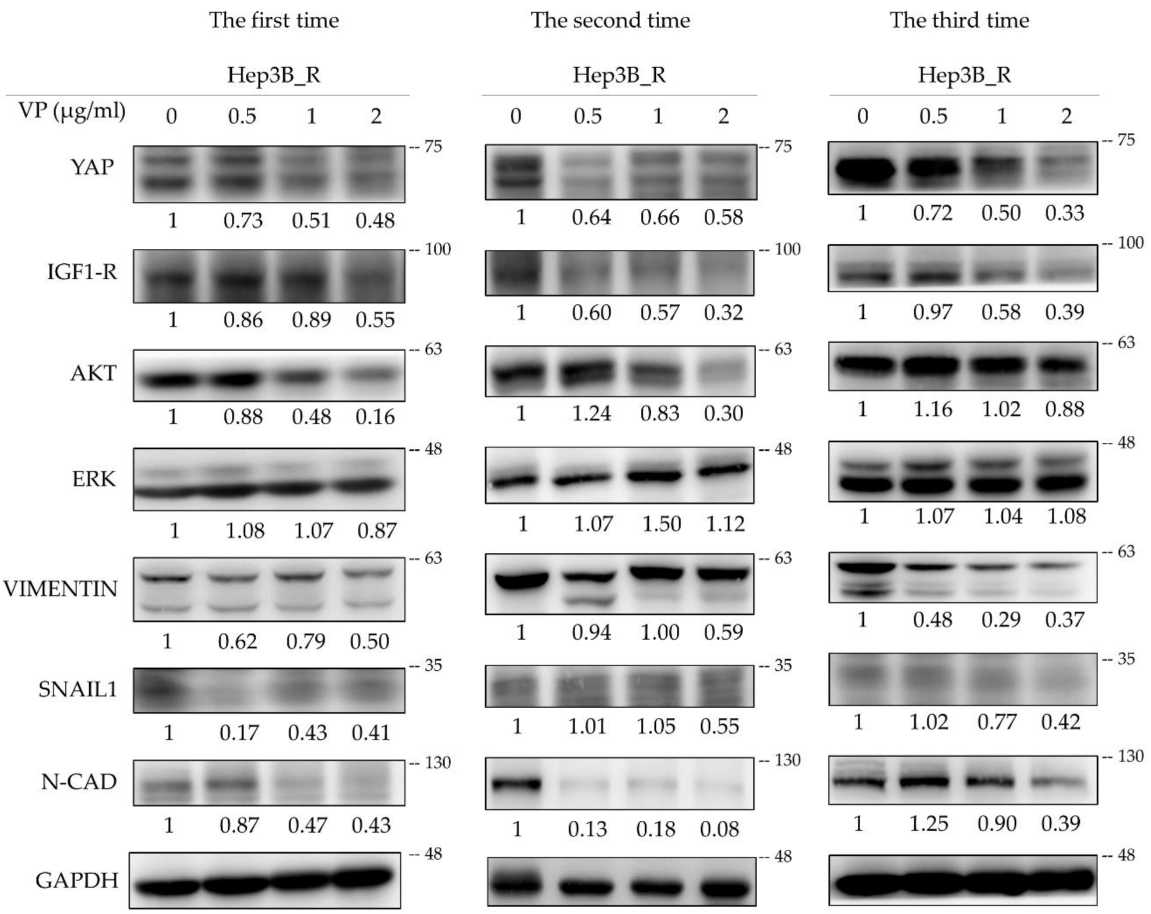

Figure S7. The triple repeats of Western blot and densitometry readings/intensity ratio of each band of Figure 3B. The effect of VP (a specific YAP inhibitor) on the expression levels of protein of YAP, IGF-1R signaling-related proteins, and the EMT-markers in Hep3B_R cells. The relative quantification was normalized to the corresponding GAPDH.

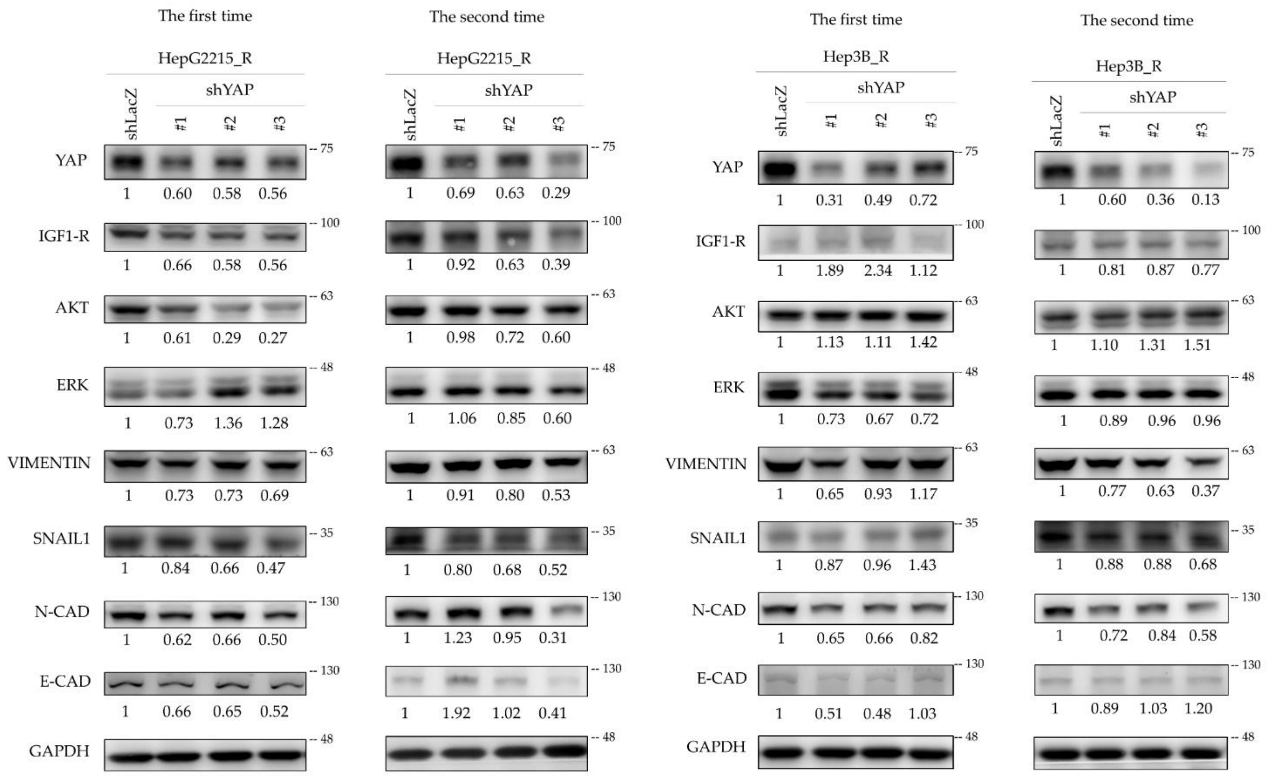

Figure S8. The double repeats of Western blot and densitometry readings/intensity ratio of each band of Figure 3D.The effect of YAP silencing by shRNA on the protein levels of YAP, IGF-1R-related signaling proteins, and EMT-markers in HepG2215-R and Hep3B_R cells. The relative quantification was normalized to the corresponding GAPDH.

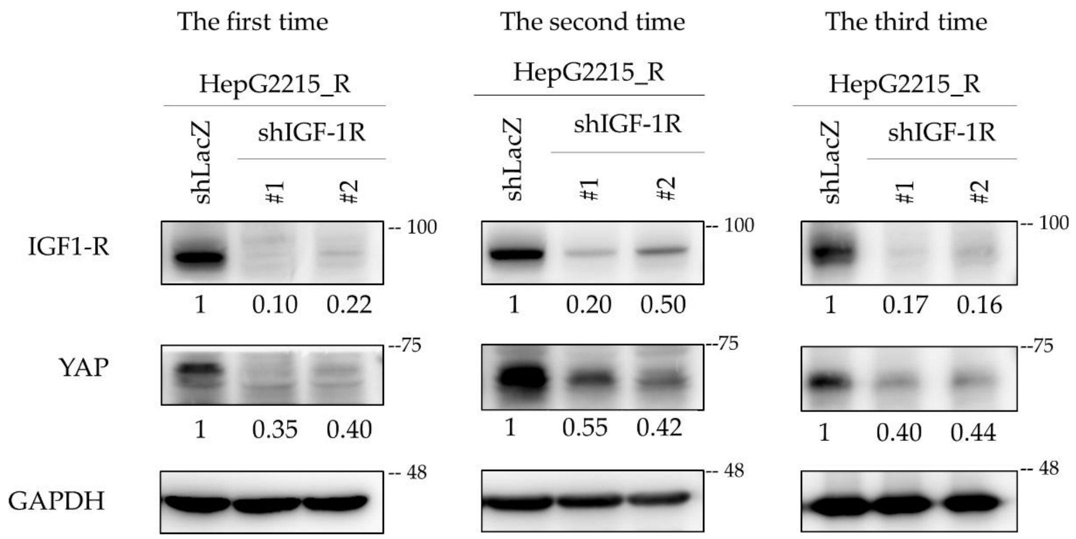

Figure S9. The triple repeats of Western blot and densitometry readings/intensity ratio of each band of Figure 4F.The effect of IGF-1R silencing by shRNA on the protein levels of YAP in HepG2215_R cells. The relative quantification was normalized to the corresponding GAPDH.

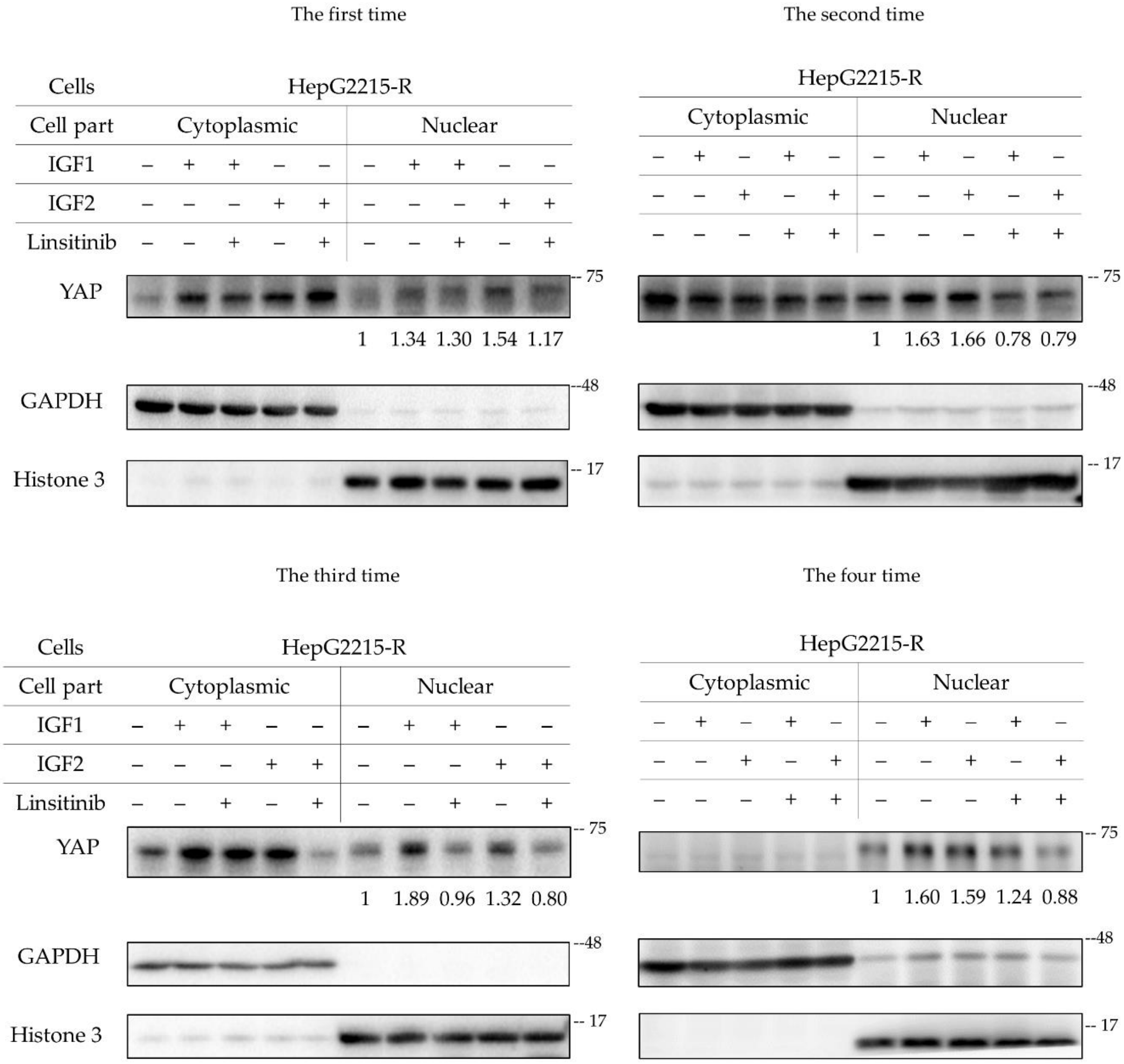

Figure S10. The four repeats of Western blot and densitometry readings/intensity ratio of each band of Figure 4H.The effect of IGF-1/2 (50 ng/ml, 1h or 2h treatment) with or without linsitinib (10 μM) on YAP expressions in cytoplasmic and nuclear fractions of HepG2215_R cells. The relative quantification was normalized to the corresponding HISTONE 3.

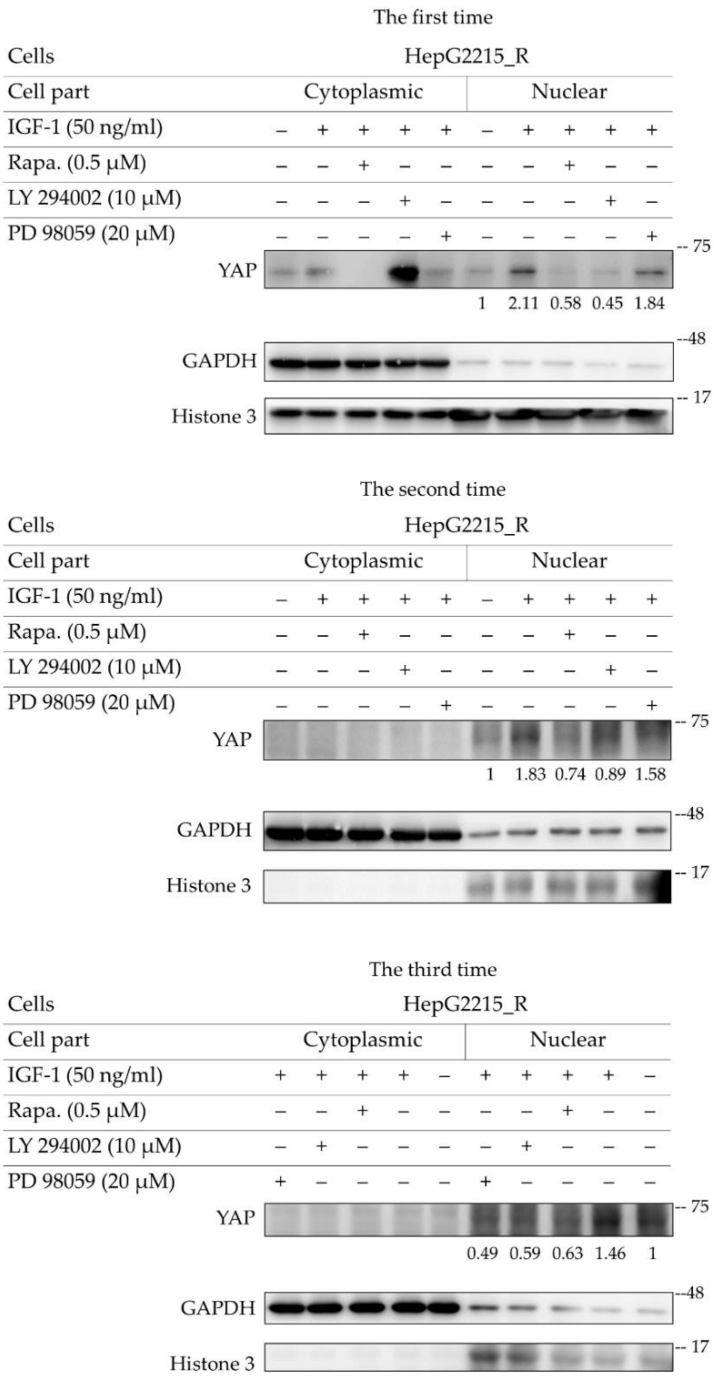

Figure S11. The triple repeats of Western blot and densitometry readings/intensity ratio of each band of Figure 4I.

The effect of signaling blockages targeting PI3K (LY294002), mTOR (Rapamycin), and Erk (PD98059) on the YAP nuclear translocation of HepG2215_R cells under IGF-1/2 treatment. The relative quantification was normalized to the corresponding HISTONE 3.

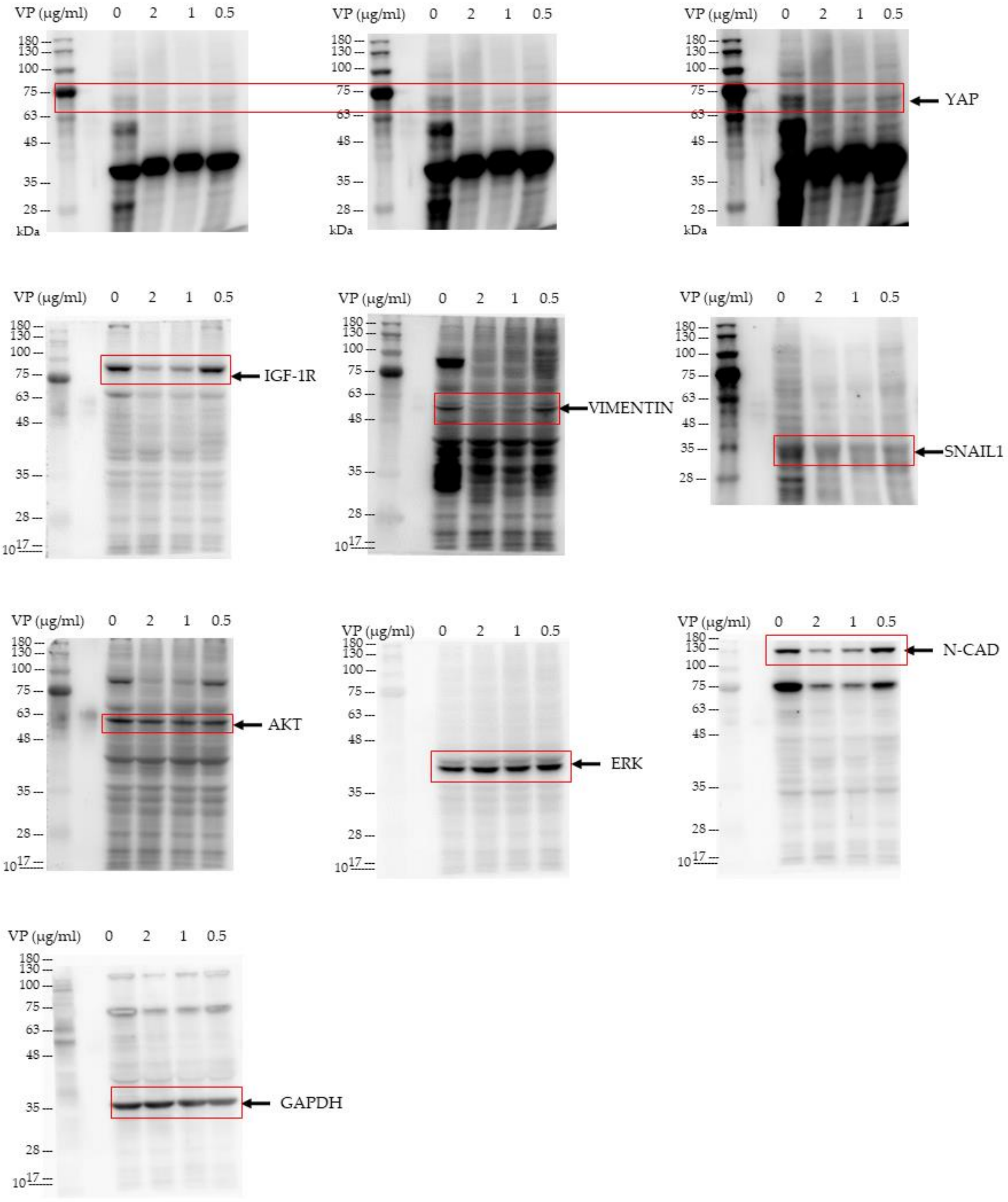

Figure S12. The full membrane Western blot data and densitometry readings/intensity ratio of each band for Figure 3B.The effect of VP (a specific YAP inhibitor) on the protein expression levels of YAP, IGF-1R signaling-related proteins, and the EMT-markers in HepG2215-R cells.

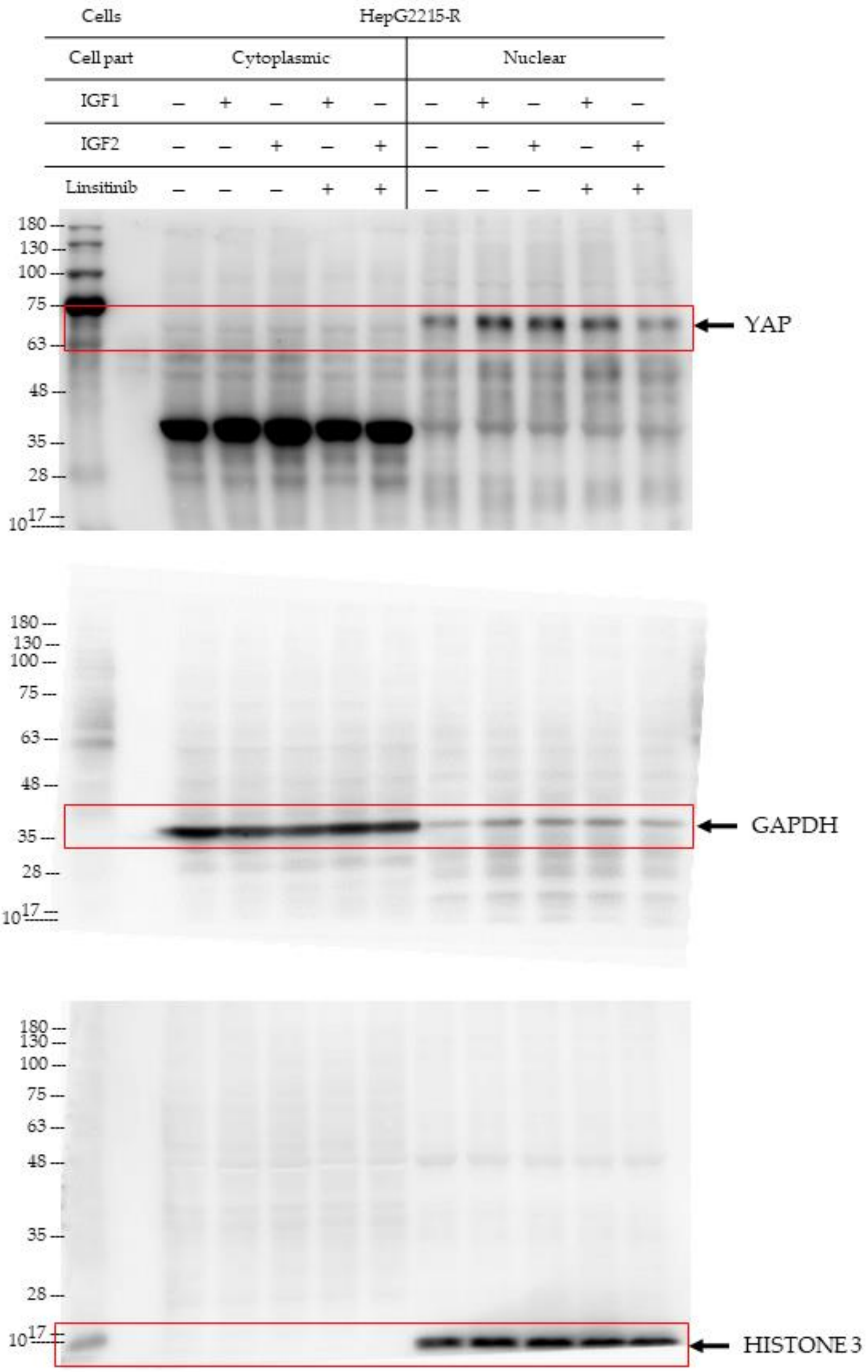

Figure S13. The full membrane Western blot data and densitometry readings/intensity ratio of each band for Figure 4H.The effect of IGF-1/2 (50 ng/ml, 1h or 2h treatment) with or without linsitinib (10 μM) on YAP expression in cytoplasmic and nuclear fractions of HepG2215_R cells were shown.

Table S1. The real-time PCR primes and product size.

Gene	Accession No.	Primer	Sequence	Product Size (bp)
YAP	NM_001282099.2	Forward	CCTTCTTCAAGCCGCCGGAG	146
		Reverse	CAGTGTCCCAGGAGAAACAGC	
IGF-1R	NM_001291858.2	Forward	CTCCTGTTTCTCTCCGCCG	84
		Reverse	ATAGTCGTTGCGGATGTCGAT	
IGF-1	NM_000618.5	Forward	AAAGAAACCTCTCACAGATAAGA	89
		Reverse	AATAATAAGGGCTGGGTTGGGAT	
IGF-2	NM_001007139.6	Forward	CACGTCCCTCTCGGACTTG	92
		Reverse	GTGGCATCGTTGAGGAGTG	
VIMENTIN	NM_003380.5	Forward	GAGAACTTTGCCGTTGAAGC	170
		Reverse	TCCAGCAGCTTCCTGTAGGT	
SNAIL1	NM_005985.4	Forward	CCCCAATCGGAAGCCTAACT	114
		Reverse	AGGATCTCCGGAGGTGGGAT	
N-CAD	NM_001308176.2	Forward	GGTGGAGGAGAAGAAGACCAG	72
		Reverse	GGCATCAGGCTCCACAGT	
E-CAD	NM_001317185.2	Forward	CCCGGGACAACGTTTATTAC	72
		Reverse	GCTGGCTCAAGTCAAAGTCC	

Table S2. List of antibodies.

Protein	Assay	Cat. No.	Company	Origin	Dilution	Incubation Period
YAP active form	WB	EPR19812	Abcam	Rabbit	1:1000	Overnight, 4 °C
YAP1 phosphorylation form S127	WB	EP1675Y	Abcam	Rabbit	1:1000	Overnight, 4 °C
p-IGF1Rβ-Tyr 1161	WB	ab39398	Abcam	Rabbit	1:1000	Overnight, 4 °C
IGF-1R	WB	SC-713	Santa Cruz Biotechnology	Rabbit	1:1000	Overnight, 4 °C
Vimentin	WB	#3932	Cell Signaling	Rabbit	1:1000	Overnight, 4 °C
Snail 1	WB	GTX125918	GENETEX	Rabbit	1:1000	Overnight, 4 °C
E-cadherin	WB	610182	BD Biosiences	Mouse	1:1000	Overnight, 4 °C
N-cadherin	WB	610921	BD Biosiences	Mouse	1:1000	Overnight, 4 °C
Akt 1/2/3	WB	SC-8312	Santa Cruz Biotechnology	Rabbit	1:1000	Overnight, 4 °C
Erk 1/2	WB	137F5	Cell Signaling Technology	Rabbit	1:4000	Overnight, 4 °C
Beta-actin	WB	SC-47778	Santa Cruz Biotechnology	Mouse	1:3000	Overnight, 4 °C
GAPDH	WB	GTX100118	GENETEX	Rabbit	1:10,000	Overnight, 4 °C
Histone	WB	17168-1-AP	Proteintech	Rabbit	1:3000	Overnight, 4 °C
YAP active form	IHC	EPR19812	Abcam	Rabbit	1:500	Overnight, 4 °C
IGF-1R	IHC	SC-713	Santa Cruz Biotechnology	Rabbit	1:100	Overnight, 4 °C
Vimentin	IHC	#3932	Cell Signaling	Rabbit	1:50	Overnight, 4 °C
Snail 1	IHC	GTX125918	GENETEX	Rabbit	1:50	Overnight, 4 °C
E-cadherin	IHC	610182	BD Biosiences	Mouse	1:100	Overnight, 4 °C
N-cadherin	IHC	610921	BD Biosiences	Mouse	1:100	Overnight, 4 °C
YAP active form	ICC	EPR19812	Abcam	Rabbit	1:500	Overnight, 4 °C

Table S3. The combination index (CI) of sorafenib (Sor.) and verteporfin (VP.).

HepG2215_R				Hep3B_R			
**Dose Sor.**	**Dose VP.**	**Effect**	**CI**	**Dose Sor.**	**Dose VP.**	**Effect**	**CI**
3.0	0.5	0.14	1.87726	3.0	0.5	0.17	1.19743
6.0	0.5	0.33	1.26569	6.0	0.5	0.27	1.1278
9.0	0.5	0.69	0.4998	9.0	0.5	0.4	0.86442
12.0	0.5	0.78	0.44145	12.0	0.5	0.46	0.87088
15.0	0.5	0.82	0.44224	15.0	0.5	0.7	0.36612
18.0	0.5	0.86	0.40935	18.0	0.5	0.8	0.24545
3.0	1.0	0.2	1.54654	3.0	1.0	0.22	1.01122
6.0	1.0	0.41	1.0281	6.0	1.0	0.35	0.82646
9.0	1.0	0.74	0.42455	9.0	1.0	0.46	0.70667
12.0	1.0	0.81	0.38995	12.0	1.0	0.55	0.61835
15.0	1.0	0.85	0.37657	15.0	1.0	0.71	0.35923
18.0	1.0	0.87	0.3885	18.0	1.0	0.86	0.15879
3.0	1.5	0.3	1.0918	3.0	1.5	0.27	0.86655
6.0	1.5	0.52	0.74991	6.0	1.5	0.43	0.62065
9.0	1.5	0.75	0.42441	9.0	1.5	0.52	0.57596
12.0	1.5	0.82	0.38032	12.0	1.5	0.66	0.39045
15.0	1.5	0.86	0.3601	15.0	1.5	0.77	0.26429
18.0	1.5	0.88	0.36644	18.0	1.5	0.92	0.0827
